# Lack of Contrast Enhancement in a Giant Perivascular Space of the Basal Ganglion on Delayed FLAIR Images: Implications for the Glymphatic System

**DOI:** 10.2463/mrms.ci.2016-0114

**Published:** 2017-01-25

**Authors:** Shinji Naganawa, Toshiki Nakane, Hisashi Kawai, Toshiaki Taoka

**Affiliations:** 1 Department of Radiology, Nagoya University Graduate School of Medicine, 65 Tsurumai-cho, Shouwa-ku, Nagoya, Aichi 466-8550, Japan

**Keywords:** magnetic resonance imaging, perivascular space, gadolinium, glymphatic system

## Introduction

### Clinical image

**Fig 1.**
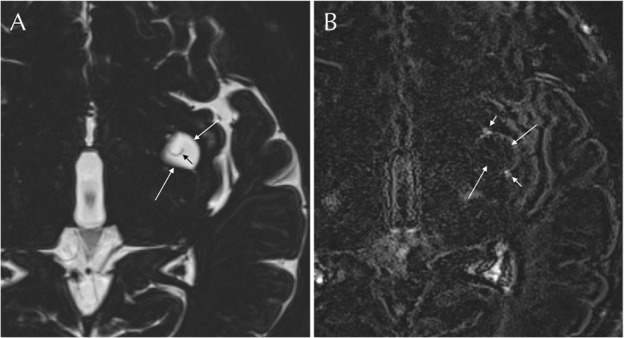
(**A**) Heavily T_2_-weighted magnetic resonance (MR) cisternography (TR/TE = 4400 ms/543 ms, 1 mm thick) shows the sharply delineated structure of the cerebrospinal fluid signal (long white arrows). A small vessel can be seen in the center of this lesion (short black arrow). This fluid signal structure is presumed to be a giant perivascular space (PVS). The size is 14.4 mm × 13.3 mm. (**B**) Heavily T_2_-weighted three dimensional-Fluid attenuated inversion recovery (3D-FLAIR) (TR/TE/TI = 9000 ms/543 ms/2250 ms, 1 mm thick) obtained four hrs after intravenous single dose administration of gadolinium based contrast agent (IV-SD-GBCA). The giant PVS does not show contrast enhancement (long white arrows), however a small nearby PVS does (short white arrows).

A 57-year-old woman with left-sided tinnitus and vertigo attacks was evaluated by magnetic resonance (MR) imaging for the presence of endolymphatic hydrops. MR imaging was performed four hrs after the intravenous single dose administration (0.1 mmol/kg) of gadolinium-based contrast agent (IV-SD-GBCA), Gadobutrol (Gadovist, Bayer HealthCare, Osaka, Japan). Heavily T_2_-weighed MR cisternography and heavily T_2_-weighted three dimensional-Fluid attenuated inversion recovery (3D-FLAIR) were obtained at 3T using previously reported methods.^1^ The estimated glomerular filtration rate (eGFR) of this patient was 75.8 mL/min/1.73 m^2^. Heavily T_2_-weighted 3D-FLAIR is quite sensitive and can detect the very low concentrations of GBCA within fluids and less sensitive to flow artifact.^2^ In the left cochlea, significant endolymphatic hydrops was observed. In addition, a large, oval, well-defined fluid signal lesion was detected in the patient’s left basal ganglion by MR cisternography. The maximum size of the lesion was 14.4 × 13.3 mm on an axial image. For reference, we also analyzed images from a routine MR examination of the brain performed six months prior at a previous hospital. The oval-shaped lesion was similar in size and had similar cerebrospinal fluid (CSF) signals on pre-contrast T_1_-, T_2_-weighted and FLAIR images. A vessel was observed in the center of the lesion. This lesion was presumed to be an extremely enlarged Virchow-Robin space or giant perivascular space (PVS). The lesion showed a CSF signal on MR cisternography and a very weak signal on heavily T_2_-weighted 3D-FLAIR obtained four hrs after IV-SD-GBCA (Fig. 1A, B). Surrounding the oval lesion were small high signal spots that appeared to be smaller PVSs based on findings from the images of upper and lower slices. The lack of enhancement suggests that the giant PVS is isolated from the CSF space.

Recently, it has been reported that PVSs in the basal ganglia show contrast enhancement on heavily T_2_-weighted 3D-FLAIR images obtained four hrs after IV-SD-GBCA (delayed FLAIR) by comparing the signal on heavily T_2_-weighted 3D-FLAIR images obtained before GBCA administration.^3^ This PVS enhancement was observed using either linear GBCA agent or macrocyclic agent and in healthy volunteers as well as patients with endolymphatic hydrops.

**Fig 2.**
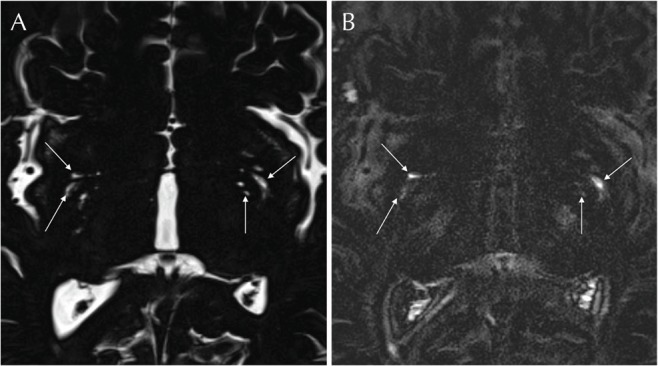
Reference images from another patient with endolymphatic hydrops (56-year-old man, estimated glomerular filtration rate (eGFR) of 64.6 mL/min/1.73 m^2^). Scan parameters were identical to those in Fig. 1. (**A**) Image from magnetic resonance (MR) cisternography shows multiple perivascular spaces in the bilateral basal ganglia. (**B**) Heavily T_2_-weighted three dimensional-Fluid attenuated inversion recovery (3D-FLAIR) obtained four hrs after intravenous single dose administration of gadolinium based contrast agent (IV-SD-GBCA). High signal intensity is observed in the bilateral perivascular spaces in the basal ganglia (arrows).

The “glymphatic system” is a recently proposed concept for waste clearance in the brain, and PVSs are the entry site to the glymphatic system. It has been proposed that CSF enters into the PVS then into the brain parenchyma and eliminates waste, such as amyloid beta, from the brain. It is thus possible that gadolinium deposition in the brain^4^ might be related to the glymphatic system.^3^ This hypothesis is further supported by the recent report that the intrathecal GBCA administration increased the signal of brain parenchyma 4.5 hrs after injection in a patient.^5^ On delayed FLAIR, CSF is usually enhanced by GBCA compared to the images obtained before GBCA administration; PVS signals are also increased, likely due to communication with the CSF space.^3^ However, the giant PVS observed in our patient does not seem to be communicating with the CSF space. Therefore, giant and normal sized PVSs might have different functional relationships with the glymphatic system (Fig. 2).

In conclusion, we report a giant PVS lacking contrast enhancement on delayed FLAIR. It is possible that this giant PVS does not have a direct connection with the CSF space unlike normal-sized PVSs.

## References

[1] Naganawa S Ohashi T Kanou M Kuno K Sone M Ikeda M. Volume quantification of endolymph after intravenous administration of a single dose of gadolinium contrast agent: comparison of 18- versus 8-minute imaging protocols. Magn Reson Med Sci 2015; 14:257–262. 25833267 10.2463/mrms.2014-0118

[2] Naganawa S. The technical and clinical features of 3D-FLAIR in neuroimaging. Magn Reson Med Sci. 2015; 14:93–106. 25833275 10.2463/mrms.2014-0132

[3] Naganawa S Nakane T Kawai H Taoka T. Gd-based contrast enhancement of the perivascular spaces in the Basal ganglia. Magn Reson Med Sci 2017; 1:61–65. 10.2463/mrms.mp.2016-0039 PMC5600045 27430361

[4] Kanda T Oba H Toyoda K Kitajima K Furui S. Brain gadolinium deposition after administration of gadolinium-based contrast agents. Jpn J Radiol 2016; 34:3–9. 26608061 10.1007/s11604-015-0503-5

[5] Eide PK Ringstad G. MRI with intrathecal MRI gadolinium contrast medium administration: a possible method to assess glymphatic function in human brain. Acta Radiol Open 2015; 4:2058460115609635. 10.1177/2058460115609635 PMC4652208 26634147

